# Effect of Extraction Conditions on the Antioxidant Activity of Olive Wood Extracts

**DOI:** 10.1155/2013/719593

**Published:** 2013-05-21

**Authors:** Mercedes Pérez-Bonilla, Sofía Salido, Adolfo Sánchez, Teris A. van Beek, Joaquín Altarejos

**Affiliations:** ^1^Departamento de Química Inorgánica y Orgánica, Facultad de Ciencias Experimentales, Universidad de Jaén, Campus de Excelencia Internacional Agroalimentario, ceiA3, 23071 Jaén, Spain; ^2^Laboratory of Organic Chemistry, Natural Products Chemistry Group, Wageningen University, Dreijenplein 8, 6703 HB Wageningen, The Netherlands

## Abstract

An investigation to optimize the extraction yield and the radical scavenging activity from the agricultural by-product olive tree wood (*Olea europaea* L., cultivar Picual) using six different extraction protocols was carried out. Four olive wood samples from different geographical origin, and harvesting time have been used for comparison purposes. Among the fifty olive wood extracts obtained in this study, the most active ones were those prepared with ethyl acetate, either through direct extraction or by successive liquid-liquid partitioning procedures, the main components being the secoiridoids oleuropein and ligustroside. An acid hydrolysis pretreatment of olive wood samples before extractions did not improve the results. In the course of this study, two compounds were isolated from the ethanolic extracts of olive wood collected during the olives' harvesting season and identified as (7′′*R*)-7′′-ethoxyoleuropein (**1**) and (7′′*S*)-7′′-ethoxyoleuropein (**2**).

## 1. Introduction

Since agricultural and industrial residues are attractive sources of natural antioxidants, basically due to their null or low value [1–4], different residues and by-products from fruits [5, 6], vegetables [7, 8], or olive oil manufacturing [9] have been screened for the presence of antioxidants. Due to the large amounts of biomass from pruning generated every year (more than 7 million tonnes per year in Spain), olive tree wood constitutes an important agricultural by-product. During the search of natural antioxidants from *Olea europaea* L. residues and by-products, both solid and liquid residues from olive oil and table olives processing have been studied [2, 10–19].

Our preliminary studies on the radical scavenging activity of olive wood extracts, cultivar Picual, showed that this agricultural by-product could be a source of natural antioxidants [20]. The isolation and radical scavenging activity of the main constituents [21] as well as some minor components present in olive wood extracts have been reported by us [22]. The secoiridoids oleuropein and ligustroside are among the main components. Other compounds present in olive wood are the lignan (+)-cycloolivil, the phenolic alcohol hydroxytyrosol, and several secoiridoids related to oleuropein, such as (7′′*S*)-7′′-hydroxyoleuropein or oleuropein 3′-*O*-*β*-D-glucoside. Moreover, the human platelet antiaggregant properties of two olive wood components, oleuropein and (+)-cycloolivil, have been evaluated [23]. The cultivar Picual was selected for these studies since it is one of the most important Spanish olive varieties for oil extraction, representing around 860.000 ha in the province of Jaén and other Andalusian areas, and is also cultivated in other regions of Spain and other countries [24].

Solvent extraction is routinely used for the isolation of antioxidants from plant material. Both extraction yield and antioxidant activity of extracts are strongly dependent on the solvent [1]. Hence, a comparative study for selecting optimal extraction conditions to provide the highest antioxidant activity (and proper extraction yield) from olive wood (cultivar Picual) was carried out in this work. For this purpose several extraction processes at room temperature and reflux were designed using solvents of different polarities. Also the influence of an acidic hydrolysis pretreatment of olive wood was investigated, since this methodology has been used sometimes to improve the recovery of phenols [3].

## 2. Materials and Methods

### 2.1. Chemicals

The solvents used for extraction (hexane, dichloromethane, ethyl acetate, *n*-butanol, ethanol, methanol, chloroform, and acetone) were glass-distilled prior to use. Methanol used for radical scavenging activity assays was of HPLC grade. Deuterated methanol was used to prepare solutions of purified compounds for NMR analysis. The 2,2-diphenyl-1-picrylhydrazyl radical (DPPH^•^, 95%) was purchased from Sigma-Aldrich Chemie (Steinheim, Germany). A commercial rosemary oleoresin was obtained from Evesa (Cádiz, Spain).

### 2.2. General Experimental Procedures

Optical rotations ([*α*]_D_) were recorded in MeOH on a Perkin-Elmer 241 automatic polarimeter (Perkin-Elmer Instruments, Norwalk, CT, USA), in a 10 cm 2 mL cell. Ultraviolet (UV) spectra were recorded in MeOH on a Perkin-Elmer UV/Vis spectrophotometer Lambda 19 (Perkin-Elmer Instruments, Norwalk, CT, USA). Infrared (IR) spectra were recorded on a FT-IR Perkin-Elmer 1760X spectrometer (Perkin-Elmer Instruments, Norwalk, CT, USA). NMR spectra (^1^H NMR, ^13^C NMR, DQF-COSY, HSQC, HMBC) were recorded on a Bruker Avance AMX 500 spectrometer (Bruker Daltonik GmbH, Rheinstetten, Germany), using CD_3_OD as solvent and tetramethylsilane (TMS) as internal reference. Mass spectra (MS) were recorded on an Finnigan MAT LCQ ion trap mass spectrometer (Waters Integrity System, Milford, MA, USA). The ESI interface was used in both positive and negative modes, with the capillary temperature at 200°C and a spray voltage of 4.5 kV.

High-performance liquid chromatography (HPLC) analyses were performed on an analytical RP-HPLC Spherisorb ODS-2 column (250 mm × 3 mm i.d., 5 *μ*m) (Waters Chromatography Division, Milford, MA, USA) on a Waters 600E instrument (Waters Chromatography Division, Milford, MA, USA) equipped with a diode array detector, scan range: 190–800 nm (Waters CapLC 2996 Photodiode Array Detector, Waters Chromatography Division, Milford, MA, USA), and operating at 30°C. Samples of the extracts were prepared in MeOH at a concentration of 10 mg mL^−1^, and the injection volume was 10 *μ*L. The best separation was performed with H_2_O : CH_3_COOH, 99.8 : 0.2, v/v (solvent A) and CH_3_OH : CH_3_COOH, 99.8 : 0.2, v/v (solvent B), at a flow rate of 0.7 mL min^−1^, using a linear gradient from 20% to 70% B for 55 min. The HPLC analyses were recorded at 230 nm, since most of the compounds present in olive extracts have an intense absorption at that wavelength.

Preparative HPLC separations were performed on an Alltima C18 column (250 mm × 22 mm i.d., 5 *μ*m) (Alltech Associates Inc., Deerfield, IL, USA) with a Shimadzu preparative HPLC instrument (Shimadzu, Kyoto, Japan), equipped with a diode array detector, scan range: 190–600 nm (SPD-M10Ap Photodiode Array Detector, Shimadzu, Kyoto, Japan) and a sample collector FRC-10 A (Shimadzu, Kyoto, Japan), and operating at 30°C and a flow rate of 12 mL min^−1^ with H_2_O : CH_3_OH : CH_3_COOH (59.9 : 39.9 : 0.2, v/v/v). 

HPLC–DAD–MS analyses were performed on an Spherisorb ODS-2 column (125 mm × 3 mm i.d., 5 *μ*m) (Waters Chromatography Division, Milford, MA, USA) with an Agilent 1100 HPLC instrument (Agilent Technologies, Santa Clara, CA, USA) equipped with a diode array detector, scan range: 190–600 nm (G1315B Photodiode Array Detector, Agilent Technologies, Santa Clara, CA, USA) and an ion trap mass spectrometer Esquire 6000 (Bruker Daltonics, Bremen, Germany). The sample preparation and gradient were the same as those in the HPLC analysis. The flow rate was 0.4 mL min^−1^. The ESI source parameters were as follows: capillary voltage: 4 kV; cap exit: –100 V; skimmer: –40 V; trap drive: 70; nebulizer: 50 psi; dry gas: 10 mL min^−1^; dry temperature: 350°C; scan range: *m/z* 50–1000.

### 2.3. Plant Material and Collection Data

Four samples of olive wood (*Olea europaea* L., cultivar Picual) were collected at two olive groves located in Jaén province (southern Spain) during the pruning period (March and April) and at the beginning of the olives' harvesting season (November) from 2003 until 2006. The samples collected were labelled as **A**, **B**, **C**, and **D**, and the location and collection date were as follows: **A** (Fuensanta village; April, 2003), **B** (Fuensanta village; March, 2005), **C** (Mogón village; March, 2005), and **D** (Fuensanta village; November, 2006). In each case, the plant material consisted of a single piece of *ca.* 10 cm diameter and 50 cm length from the pruning of the same olive grove near Fuensanta (**A**, **B**) and a different olive grove near Mogón (**C**). Another similar single piece was cut in the same olive grove near Fuensanta during the olives' harvesting season for comparison (**D**). Each sample was stored in a dry and dark place at room temperature with passive ventilation for 3 months. Just before starting the extraction process, the samples (including bark and heartwood) were scraped in a local sawmill (wood shavings: length 3–5 cm, thickness 0.1–0.3 mm).

### 2.4. Extraction Protocols

Olive wood samples **A**, **B**, **C**, and **D** were extracted by the following procedures (*i*–*iv*).

**Figure 1:**
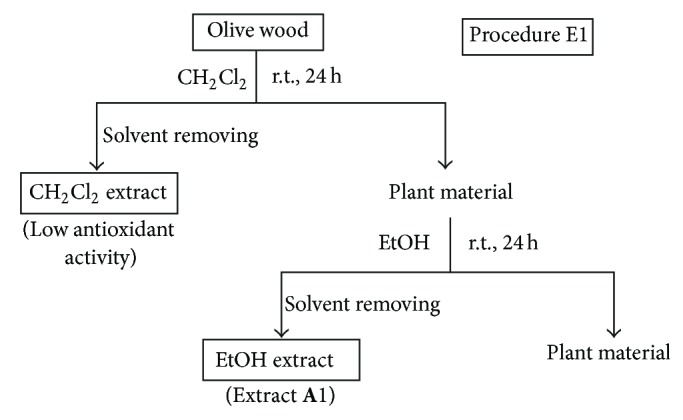
Solvent extractions of olive wood sample **A** following procedure E1.

**Figure 2:**
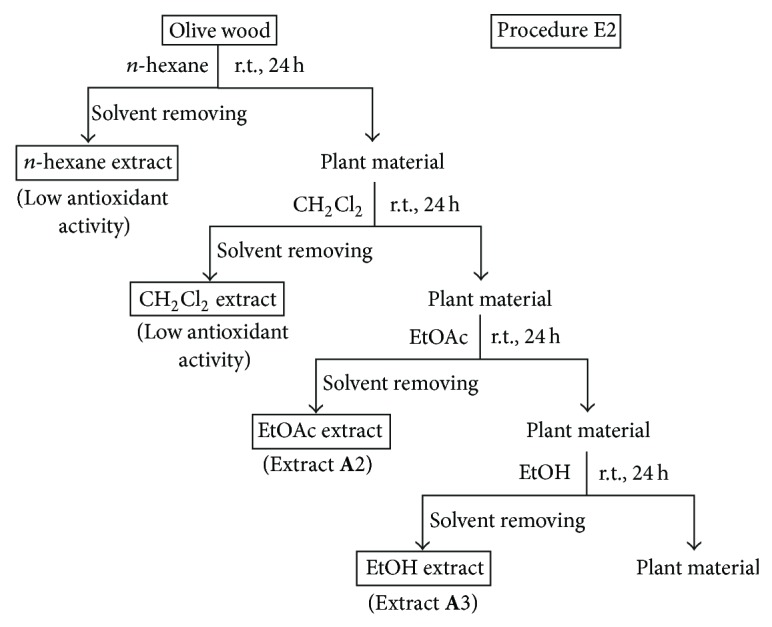
Solvent extractions of olive wood sample **A** following procedure E2.

**Figure 3:**
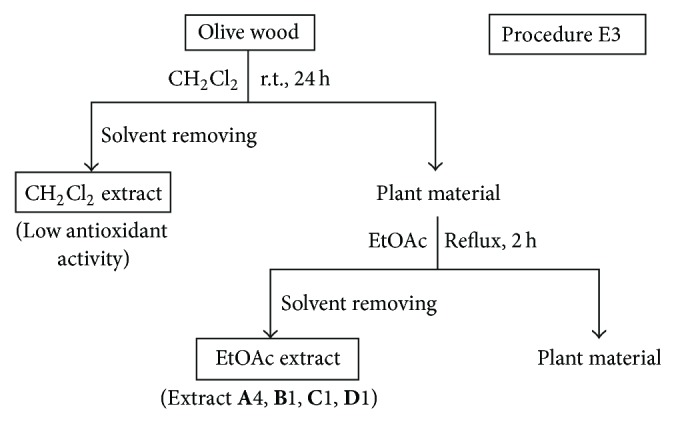
Solvent extractions of olive wood samples **A**, **B**, **C**, and **D** following procedure E3.

*(i) Procedures E1, E2, and E3.* These procedures involved the sequential extraction of olive wood samples with solvents of increasing polarity at room temperature for 24 h or under reflux for 2 h. The procedure E1 employed the sequence of solvents CH_2_Cl_2_ and EtOH at room temperature (Figure 1). The procedure E2 used the sequence of solvents *n*-hexane, CH_2_Cl_2_, EtOAc, and EtOH at room temperature (Figure 2). The procedure E3 employed CH_2_Cl_2_ at room temperature and then EtOAc under reflux (Figure 3). The olive wood sample **A** (35 g each) was extracted by the procedures E1 and E2 using 250 mL of each solvent (extracts **A**1–**A**3; see Table 2). Olive wood samples **A**, **B**, **C**, and **D** (35 g each) were extracted by the procedure E3 using 500 mL of each solvent (extracts **A**4, **B**1, **C**1, and **D**1; see Tables 2 and 4). The extracts prepared with *n*-hexane and CH_2_Cl_2_ were discarded since their radical scavenging activities were low.

**Figure 4:**
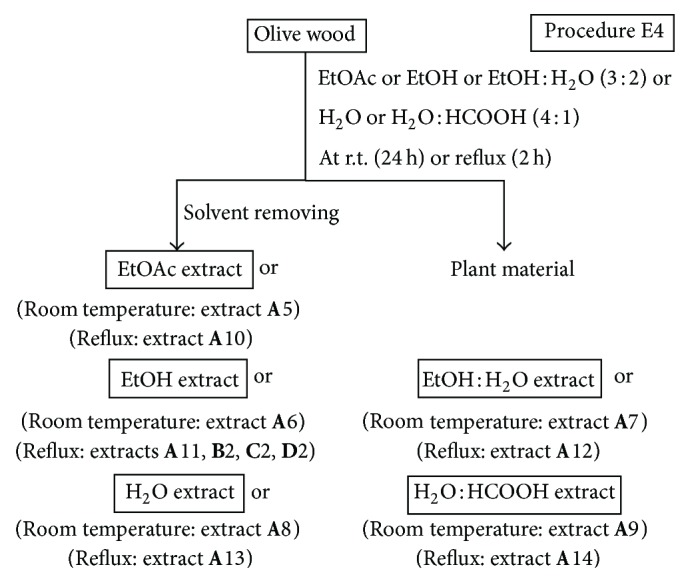
Solvent extractions of olive wood samples **A**, **B**, **C**, and **D **following procedure E4.

*(ii) Procedure E4.* This procedure involved the direct extraction of olive wood samples with different solvents at room temperature for 24 h and under reflux for 2 h (Figure 4). The solvents used were EtOAc, EtOH, EtOH : H_2_O (3 : 2, v/v), H_2_O, and H_2_O : HCOOH (4 : 1, v/v). The olive wood sample **A** (35 g) was extracted by the procedure E4 at room temperature (**A**5–**A**9) and under reflux using 500 mL of each solvent (**A**10–**A**14) (Table 2). The olive wood samples **B**, **C**, and **D** (35 g each) were extracted by the procedure E4 using 500 mL of EtOH under reflux (extracts **B**2, **C**2, and **D**2) (Table 4).

**Figure 5:**
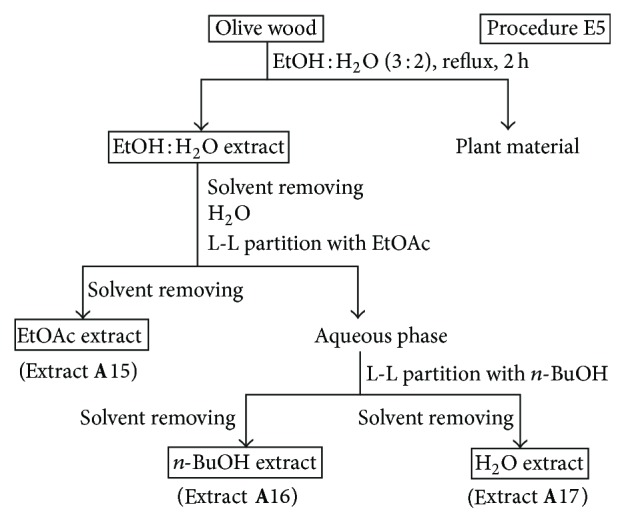
Solvent extractions of olive wood sample **A** following procedure E5.

**Figure 6:**
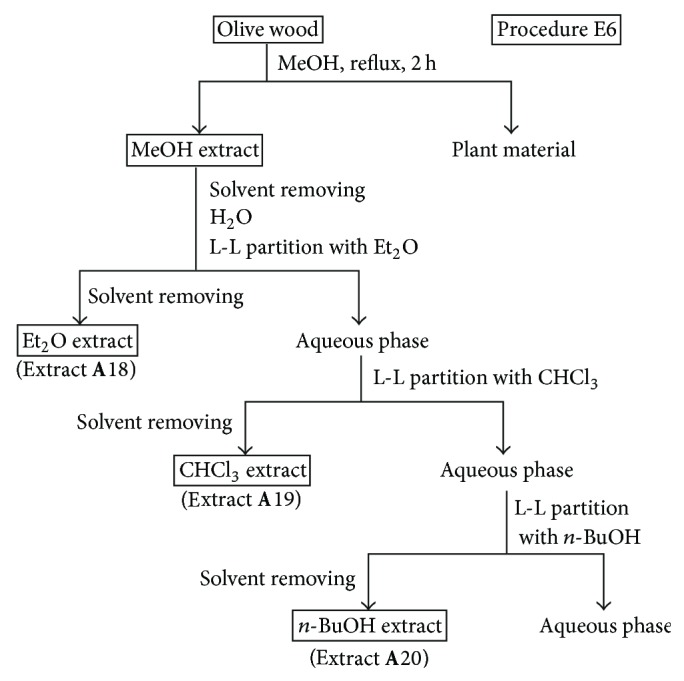
Solvent extractions of olive wood sample **A** following procedure E6.

*(iii) Procedures E5 and E6.* These procedures involved the direct extraction of olive wood samples with a polar solvent under reflux for 2 h, followed by a liquid-liquid partitioning of the resulting extract with solvents of increasing polarity (Figures 5 and 6). A mixture of EtOH : H_2_O (3 : 2, v/v) was used for the direct extraction in procedure E5 (Figure 5), while MeOH was used in procedure E6 (Figure 6). Olive wood sample **A** (30 g) was extracted by the procedure E5 using 350 mL of EtOH : H_2_O (3 : 2). The resulting EtOH : H_2_O extract was evaporated to dryness under vacuum, suspended in water (200 mL), and successively liquid-liquid partitioned with EtOAc (150 mL) and *n*-BuOH (150 mL) to yield extracts **A**15 and **A**16, respectively (Figure 5 and Table 2). The remaining aqueous phase was also evaporated to yield extract **A**17. Another olive wood sample **A** (30 g) was extracted by the procedure E6 using 350 mL of MeOH. In a similar manner, the resulting MeOH extract was evaporated and partitioned with Et_2_O (150 mL), CHCl_3_ (150 mL), and *n*-BuOH (150 mL) to yield extracts **A**18, **A**19, and **A**20, respectively (Figure 6, Table 2).

**Figure 7:**
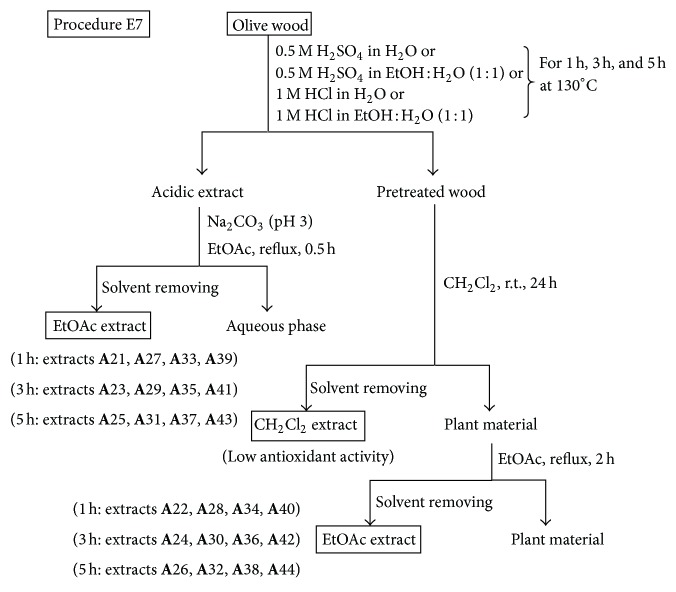
Hydrolysis pretreatment and solvent extractions of olive wood sample **A** following procedure E7.

*(iv) Procedure E7.* This procedure involved an acidic hydrolysis pretreatment of the olive wood samples using different acids for 1 h, 3 h, and 5 h at 130°C, followed by a solvent extraction of both the resulting liquid acidic extract and solid pretreated wood (Figure 7). Every olive wood sample **A** (5 g each) was hydrolysed with 40 mL of 0.5 M H_2_SO_4_ in H_2_O, 40 mL of 0.5 M H_2_SO_4_ in EtOH : H_2_O (1 : 1, v/v), 40 mL of 1 M HCl in H_2_O, or 40 mL of 1 M HCl in EtOH : H_2_O (1 : 1, v/v) under the conditions described above. Thus, for example, an olive wood sample **A** (5 g) was pretreated with 40 mL of 0.5 M H_2_SO_4_ in H_2_O for 1 h at 130°C. Then, the mixture was filtered, and the liquid acidic extract diluted with water (200 mL) and its pH adjusted to 3 with Na_2_CO_3_. EtOAc (100 mL) were added to the acidic aqueous phase and refluxed for 0.5 h. Then, the EtOAc layer was separated and dried over anhydrous Na_2_SO_4_, and the EtOAc extract evaporated to dryness under vacuum to yield extract **A**21 (Figure 7 and Table 3). This procedure generated, after the initial filtering, a solid pretreated wood, which was also extracted first with CH_2_Cl_2_ at room temperature for 24 h and then with EtOAc under reflux for 2 h (Figure 7). The CH_2_Cl_2_ extract was discarded, due to its low radical scavenging activity, and the EtOAc extract **A**22 was kept (Figure 7 and Table 3). Another olive wood sample **A** (5 g) was pretreated with 40 mL of 0.5 M H_2_SO_4_ in H_2_O for 3 h at 130°C, yielding finally extracts **A**23 and **A**24 (Figure 7 and Table 3). Since the hydrolysis pretreatments of olive wood samples were carried out with five different acid conditions and three different times (1 h, 3 h, and 5 h) and each pretreatment yielded two extracts to be investigated, the procedure E7 afforded twenty-four extracts (Figure 7 and Table 3).

The solvent of the extracts obtained in the different procedures was evaporated under vacuum at temperatures not higher than 40°C. The resulting dry extracts were stored under argon in sealed vials at −20°C until analysis. Extraction yields were calculated as grams of the dry extract per kilogram of olive wood sample.

### 2.5. DPPH Radical Scavenging Assay

Radical scavenging activity of extracts was determined spectrophotometrically with the stable DPPH radical [25, 26]. Methanolic solutions (2.4 mL) of DPPH^•^ (~7 × 10^−5 ^mol L^−1^) with an absorbance at 515 nm of 0.80 ± 0.03 AU were mixed with methanolic solutions (1.2 mL) of extracts at 50 *μ*g mL^−1^ by dissolving the dry extracts in methanol. The experiment was carried out in triplicate. The samples were shaken and kept in the dark for 15 min at room temperature, and then the decrease of absorbance was measured at 515 nm. Radical scavenging activity of extracts is expressed as radical scavenging percentage (RSP) and was calculated using the following equation [26]:

(1) RSP(%)=[AB−AAAB]×100,

where *A*_*B*_ is the absorbance of the blank (*t* = 0 min) and *A*_*A*_ is the absorbance of the tested extract solution (*t* = 15 min). 

### 2.6. Isolation and Structure Elucidation of Purified Compounds

An aliquot (73 mg) of extract **D**2 (Figure 4 and Table 4) was chromatographed by preparative RP-HPLC (see Section 2.2) to afford compounds **1** and **2**. Pure compounds **1** (21 mg) and **2** (15 mg) were obtained after removing the solvents with a rotary evaporator and the remaining H_2_O with a freeze dryer. The structures of purified compounds were elucidated by various spectroscopic methods and specific optical rotations measurements (see Section 2.2).

*(7* ′′ *R)-7* ′′ *-Ethoxyoleuropein *(**1**). Colourless syrup; [*α*]_D_^25^–112° (*c* 0.10, methanol); UV (methanol) *λ*_max_ (log *ε*) 231 (4.04), 282 nm (3.36); IR (film) *ν*_max_ 3384 (OH), 1705 (C=O), 1629 (*α*,*β*-unsaturated C=O), 1384, 1076, 1045 (C–O–C) cm^−1^; ESIMS (positive), *m/z* 607.2 ([M+Na]^+^), and 1190.7 ([2 M+Na]^+^), ESIMS (negative), *m/z* 583.2 ([M–H]^−^); for ^1^H and ^13^C NMR data see Table 1.

*(7* ′′ *S)-7* ′′ *-Ethoxyoleuropein *(**2**). Colourless syrup; [*α*]_D_^25^–95° (*c* 0.05, methanol); UV (methanol) *λ*_max_ (log *ε*) 231 (4.17), 282 nm (3.50); IR (film) *ν*_max_ 3385 (OH), 1703 (C=O), 1628 (*α*,*β*-unsaturated C=O), 1384, 1076, 1045 (C–O–C) cm^−1^; ESIMS (positive), *m/z* 607.2 ([M+Na]^+^), and 1190.8 ([2M+Na]^+^), ESIMS (negative), *m/z* 583.3 ([M–H]^−^); for ^1^H and ^13^C NMR data see Table 1. 

## 3. Results and Discussion

Following up our preliminary results on the radical scavenging activity of dichloromethane and ethanol extracts of olive (*O. europaea*) wood [20], several extraction procedures using different sequences of solvents with different polarities, at different temperatures and times were investigated in this work. Moreover, the influence of using both (a) acidified solvents (e.g. mixture of water and formic acid) to extract olive wood and (b) acidic hydrolysis pretreatments of the plant material on the yield and antioxidant activity of the resulting extracts was also studied (Figures 1–7). Four samples of olive tree wood, cultivar Picual, have been used to prepare all the extracts; three of them were collected in the same olive grove during the pruning period (**A**, **B**) or the harvesting season (**D**) and the other one in a different olive grove (**C**). All extracts obtained have been evaluated for their radical scavenging activities, except the hexane and dichloromethane extracts. The two latter ones showed low antioxidant activity in our previous works but allowed the removal of nonpolar components from polar extracts [20, 21]. Tables 2–4 show the extraction yields and the DPPH radical scavenging activity of the fifty extracts obtained in this work. The purpose of this work was to find improved extraction conditions to yield olive wood extracts with high antioxidant activities and appropriate extraction yields. The design of every extraction procedure was based on our previous experience and that of others working on optimization of extraction processes, taking into account general considerations on cost, easiness, and suitable scaling-up. Thus, procedure E1 involved a simple sequential extraction with dichloromethane and ethanol at room temperature (Figure 1). This protocol was used in our preliminary work [20] and allowed us to conclude that ethanol yielded the largest amounts of extracts, with the highest radical scavenging activities. Procedure E2 used the sequence of solvents *n*-hexane, dichloromethane, ethyl acetate, and ethanol at room temperature (Figure 2) in order to perform a better separation of metabolites by groups and choose the better solvent (ethyl acetate or ethanol) to extract antioxidants. As ethyl acetate seemed to be a more selective solvent to extract antioxidants from olive wood, procedure E3 used only two solvents to simplify the protocol (Figure 3): dichloromethane at room temperature to remove nonpolar components, and ethyl acetate under reflux to increase extract yields in active compounds. Procedure E4 involved the direct extraction of olive wood with ethyl acetate at room temperature or under reflux, without previous removal on nonpolar components, and the direct extraction with several more polar solvents, including acidified water (water-formic acid), for comparison purposes (Figure 4). Procedures E5 and E6 involved the initial extraction under reflux of olive wood with aqueous ethanol (Figure 5) and methanol (Figure 6), respectively, followed by several liquid-liquid partitioning with solvents of increasing polarity. These “inverse” procedures were designed to compare results with those of the direct extraction in sequence included in procedures E1, E2, and E3. Finally, procedure E7 (Figure 7) was designed to explore the influence on yield and antioxidant activity of the extracts obtained after an acidic treatment of olive wood (see the following). 

### 3.1. Temperature Effect

The influence of temperature on the extraction was investigated since it affects both the equilibrium and mass transfer rate. Higher temperatures could produce the breakage of bonds between analytes and plant matrix and could thus increase the yield of the extraction [27] or could favour the reaction of compounds like phenols with other plant components, impeding their extraction [1]. Higher temperatures increase the solubility of the compounds, although they may also affect their stability, and chemical transformations may happen; the changes in extract composition usually involve changes in radical scavenging activity. In this study, olive wood shavings from sample **A** were subjected to extraction with solvent systems at two temperatures, room temperature, and reflux temperature of each solvent (see Section 2), and as expected, higher yields were obtained under reflux (from 11.4 to 211.4 g extract kg^−1^ wood) than at room temperature (from 8.6 to 122.9 g extract kg^−1^ wood) (Table 2). For instance, the increase of the yield of extracts obtained under reflux with respect to those obtained at room temperature is considerable; from a 30% (extract **A**4 versus extract **A**2) up to a 136% (extract **A**11 versus extract **A**6). In contrast, the increases observed for the radical scavenging percentage (RSP) of the same extracts are comparatively lower: from a 3% (extract **A**4 versus extract **A**2) up to a 24% (extract **A**11 versus extract **A**6). In general, it can be said that extract yields reach a considerable increase with the temperature while RSP values increase moderately. Indeed, the highest value for radical scavenging activity at room temperature (63.2%, extract **A**2) was similar to the highest one for extractions under reflux (64.9%, extract **A**4) (Table 2). This means that the extraction protocols using refluxing solvents are not necessarily better than those using solvents at room temperature.

### 3.2. Solvent Composition

Since the radical scavenging activity depends on the extract composition, comparative studies for selecting the optimal solvents providing maximum antioxidant activity are required for each plant material. Methanol, mixtures of ethanol (or methanol) and water, ethyl acetate, and diethyl ether have been the most common extraction solvents reported in the literature for the extraction of phenols from wood samples [28]. Considering our previous work on olive wood extracts [20–22], where nonoptimized extraction protocols were used, a more comprehensive study of the extraction yields and radical scavenging activities of olive wood extracts obtained from sample **A** with different neat solvents or mixtures of them was carried out in this work (Figures 1–6). The results are shown in Table 2. Hexane, dichloromethane, ethyl acetate, and ethanol were chosen as solvents to extract olive tree wood shavings in three different manners (procedures E1, E2, and E3). The ethyl acetate extract obtained under reflux of an olive wood sample (**A**4), previously extracted with dichloromethane at room temperature, afforded the best radical scavenging activity (64.9%). Ethyl acetate, ethanol, ethanol-water, water, and water-formic acid mixtures have also been used for each extraction of a fresh and non previously extracted olive tree wood sample, following procedure E4 (Figure 4). Ethanolic and aqueous extracts yields were around 6-fold and 9-fold higher than those of ethyl acetate extracts, respectively (Table 2). It is documented that an addition of water to solvents causes swelling of the plant material, thereby allowing the solvent to penetrate the solid matrix more easily, which leads to higher yields [27]. However, the radical scavenging percentages of ethanolic and aqueous extracts were up to 3-fold lower than those of ethyl acetate extracts. Moreover, a pH effect on the extraction yield has also been reported [1]. To study the pH influence, two additional extracts obtained with a water-formic acid mixture have also been evaluated in this work (extracts **A**9 and **A**14). The yields of these extracts were higher than those of aqueous extracts without acid (extracts **A**8 and **A**13), but there were no significant changes in their radical scavenging percentages. Two other extraction procedures (E5 and E6) have been checked according to those described in the literature [29, 30]. In both procedures, an aqueous ethanolic extract (procedure E5, Figure 5) and a methanolic extract (procedure E6, Figure 6) were partitioned using successively ethyl acetate and *n*-butanol (procedure E5) and diethyl ether, chloroform, and *n*-butanol (procedure E6) as solvents. The yields of alcoholic (extracts **A**16 and **A**20) and aqueous extracts (extract **A**17) were the highest ones, and the ethyl acetate extract (extract **A**15) was the most active one again. Thus, ethyl acetate extracts from olive wood, obtained either through direct extraction (**A**4) or by successive liquid-liquid partitioning (**A**15) procedures, are the most active ones. This solvent has been used on other occasions to separate low molecular weight polyphenols from other wood sources [31, 32].

### 3.3. Acid Hydrolysis Pretreatment Effect on Wood Shavings

It is known that during the conversion of hemicellulose into sugar and sugar oligomers by mild hydrolysis of lignocellulosic materials, cell wall-linked phenolic compounds are also solubilized [12]. A number of technologies are available for the hydrolysis of these materials. The autohydrolysis is the simplest one, where the lignocellulosic material is contacted with water or steam [3, 33]. In hardwoods, acid hydrolysis processes have been extensively studied as well as the effect of the operational conditions on the yield and the antioxidant activity of the phenolic fraction recovered [3]. Solvent extraction with ethyl acetate has successfully been applied for purification or refining purposes, since saccharides remain in the aqueous phase, whereas the nonsaccharide compounds (part of them, of phenolic nature) are transferred to the organic phase. To evaluate the effect of this pretreatment on olive wood samples, several experiments were designed based on the literature using sulfuric acid [34, 35] or hydrochloric acid [36]. In this work, after acidic hydrolysis pretreatment of olive wood shavings, the filtration and extraction of both liquid and solid phases were carried out using procedure E7 (Figure 7). Table 3 shows the extraction yields and the DPPH radical scavenging activity of the ethyl acetate extracts obtained according to this procedure. Yields of the ethyl acetate extracts obtained from the liquid phase of procedure E7 (“acidic extract” in Figure 7) by partitioning against ethyl acetate (extracts **A**21, **A**23, **A**25, **A**27, **A**29, **A**31, **A**33, **A**35, **A**37, **A**39, **A**41, **A**43) were up to 10-fold higher than those of ethyl acetate extracts obtained following a consecutive extraction of the solid phase of procedure E7 (“pretreated wood” in Figure 7) with dichloromethane and ethyl acetate (extracts **A**22, **A**24, **A**26, **A**28, **A**30, **A**32, **A**34, **A**36, **A**38, **A**40, **A**42, and **A**44) (Table 3). However, among the radical scavenging percentage data of the latter extracts (from 29.9 to 68.7%), the best result (extract **A**36, 68.7%) was only 10% higher than the highest one (extract **A**35, 58.5%) of the former ones (from 41.6 to 58.5%). Besides, it can be said that there are no important differences between the radical scavenging activities obtained with the acidic pretreatment of olive wood using sulfuric acid (63.1% is the highest radical scavenging value; extract **A**32) or hydrochloric acid (68.7% is the highest one; extract **A**36), neither in aqueous-alcoholic solutions nor in aqueous solutions. In terms of pretreatment time, the radical scavenging activity was in general higher after 3 or 5 h than after 1 h. In conclusion, the best radical scavenging activities were those corresponding to extracts **A**36 and **A**38, obtained from the ethyl acetate extraction of the pretreated olive wood shavings with 1 M HCl after 3 h and 5 h, whose radical scavenging percentages were 68.7% and 64.5%, respectively. These results were similar to that obtained for extract **A**4 (64.9% of radical scavenging activity), which was prepared by extraction with ethyl acetate under reflux of the same wood sample **A** previously extracted with dichloromethane according to procedure E3 (Figure 3 and Table 2). However, if yield values are compared, it can be said that the acid hydrolysis pretreatment of olive wood does not improve the simplest and cheapest procedure E3, since extract **A**4 was obtained with a 14.2% yield while extracts **A**36 and **A**38 were obtained with 6.1% and 9.0% yields, respectively.

### 3.4. Location and Season Collection Data

**Figure 8:**
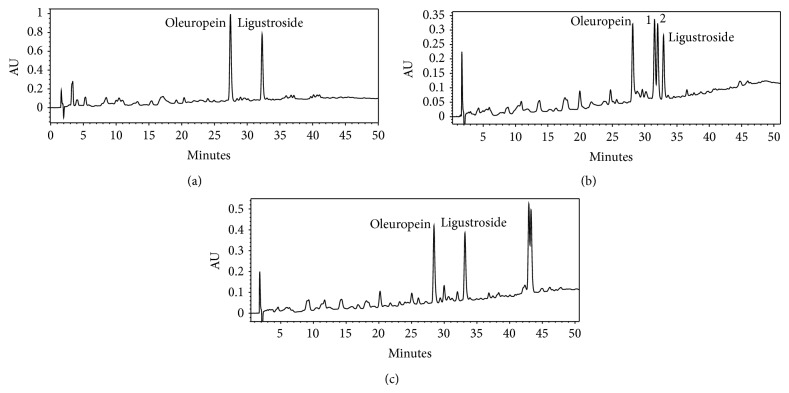
HPLC profiles of olive wood extracts at 230 nm: (a) ethyl acetate extract **A**4, (b) ethanol extract **D**2, and (c) direct *n*-butanol extract from olive wood sample **D**.

The geographic origin, as well as climatic condition, harvesting date, storage, environmental, and technological factors, affects the composition of plant material samples and consequently their antioxidant activities [1, 24, 37]. Four different woods collected at two different locations and seasons have been studied (samples **A**, **B**, **C**, and **D**; see Section 2 for details). Taking into consideration the best results previously found for sample **A**, procedure E3 was chosen as the extraction protocol for extracting the other three samples. Later, these samples were also extracted by procedure E4 (with EtOH under reflux), which also showed good behaviour with sample **A**. Yields of the ethyl acetate extracts (procedure E3) ranged from 14.2 g extract kg^−1^ wood for **A** to 91.8 g extract kg^−1^ wood for **C**, and the yields of the ethanol ones (procedure E4) from 81.7 g extract kg^−1^ wood for **D** to 172.5 g extract kg^−1^ wood for **C** (Table 4). Hence, yields of the ethyl acetate extracts were lower than those of the ethanol ones which is in agreement with previous results obtained for sample **A**. However, some differences in antioxidant activity and composition have been found among the four olive wood samples studied; regarding the radical scavenging activity, the ethyl acetate extract of the sample collected during the olives' harvesting season (sample **D**) showed a slightly lower radical scavenging percentage than that of the ethanol extract, which is in contrast with the results obtained for sample **A**. The HPLC–DAD–MS analyses of seven of these eight extracts (both ethyl acetate and ethanol extracts) showed similar chromatographic profiles (Figure 8(a)), where oleuropein and ligustroside were the main components as described before by us for other olive wood samples [21, 22]. However, the chromatogram of the ethanolic extract from the sample collected during the olives' harvesting season (extract **D**2) showed four major peaks: oleuropein, ligustroside, and two compounds not identified previously by us (Figure 8(b)). 

### 3.5. Structure Elucidation of the Unidentified Compounds

**Scheme 1:**
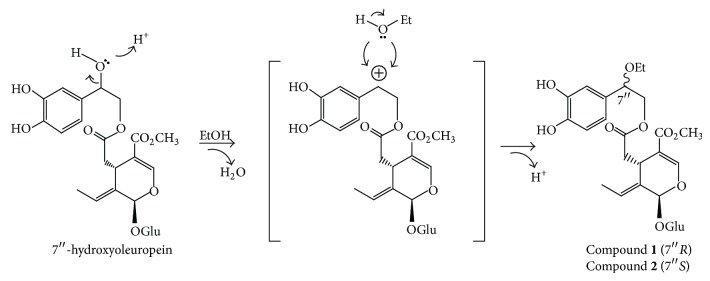
Proposed reaction pathway for the conversion of the natural product 7′′-hydroxyoleuropein into the artefacts **1** and **2** during the extraction of olive wood (sample **D**) with ethanol.

**Figure 9:**
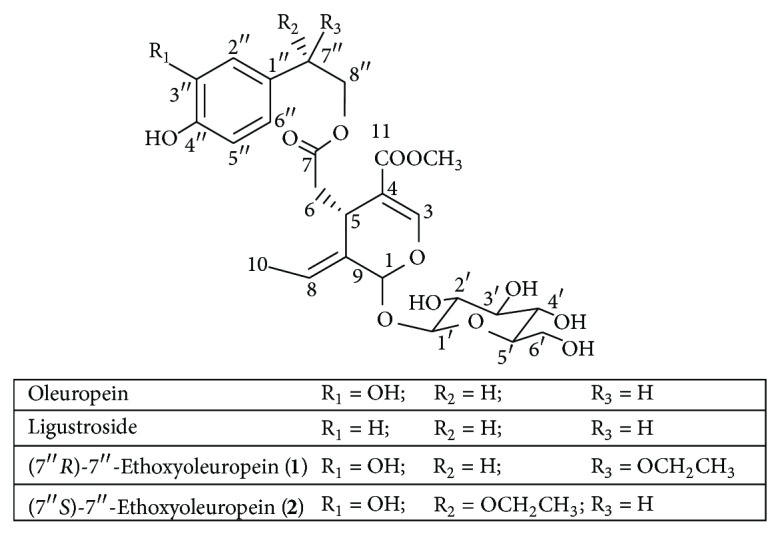
Structures of the isolated compounds from the olive wood sample **D**.

An aliquot of the ethanolic extract from the sample collected in autumn (extract **D**2) was submitted to further preparative RP-HPLC separations, and two pure secoiridoids, compounds **1** and **2**, were therefore isolated (Figure 9). Compounds **1** and **2** were characterized by UV, IR, MS, ^1^H NMR, ^13^C NMR, 2D NMR, and specific optical rotation measurements. These spectroscopic data indicate that compounds **1** and **2** were two stereoisomers of 7′′-ethoxyoleuropein. The spectral data of **1** are in agreement with earlier published data for lucidumoside C, which was isolated for the first time from an ethanolic extract of *Ligustrum lucidum* fruit [38]. However, the exact configuration at C-7′′ was not given in that paper. In order to establish the stereochemistry at C-7′′ of **1** and **2**, a comparative study of the NMR spectra of both compounds with those of (7′′*R*)- and (7′′*S*)-7′′-methoxyoleuropein was carried out. These methoxyoleuropein derivatives were isolated for the first time from the methanolic extract of *Jasminun officinale* L. var. *grandiflorum* leaves and stems [39]. The 7′′*R*-epimer has signals for H-8′′a and H-8′′b differing by 0.06 ppm while in the 7′′*S*-epimer the signals for H-8′′a and H-8′′b differ by 0.20 ppm. Since compounds **1** and **2** showed Δ*δ* values of 0.03 and 0.17, respectively, the stereochemistry at carbon C-7′′ for **1 **is assigned as 7′′*R* and for **2** as 7′′*S*. Both diastereoisomers of 7′′-ethoxyoleuropein seem to be artefacts of 7′′-hydroxyoleuropein produced by the extraction with ethanol. In order to prove this hypothesis, the same olive wood sample **D** was extracted following the same extraction procedure (procedure E4; 2 h at reflux) with acetone, in one case, and with *n*-butanol in the other case. The corresponding acetone and *n*-butanol extracts were analysed by HPLC–DAD–MS and only the second extracts presented two new peaks with an [M–H]^−^ ion at *m/z* 611.2 for the two diastereoisomers. These were assigned as the corresponding artefacts produced by *n*-butanol (Figure 8(c)), although no further efforts were made to isolate them. It is well documented that olive drupes contain a hydroxylated oleuropein derivative, with a hydroxyl group at the elenoic moiety, known as 10-hydroxyoleuropein [40]. However, the presence of 7′′-hydroxyoleuropein, with the hydroxyl group located at the phenylethanolic moiety, has only been detected in some occasion in olive drupes [41]. Recently, we reported the presence of (7′′*S*)-7′′-hydroxyoleuropein in olive wood [22]. This molecule, never found previously in *O. europaea*, is a secondary metabolite in other genera of the Oleaceae family, such as *Fraxinus* and *Ligustrum* [42]. It is known that a hydroxyl group located at a benzylic position, such as in the case of 7′′-hydroxyoleuropein, is endowed with a special reactivity. Indeed, the acid-catalysed synthesis of ethers from benzylic alcohols and aliphatic alcohols has been described [43]. Thus, we can postulate that when the olive wood extracts were prepared under reflux with ethanol as solvent, a catalytical substitution of the hydroxyl group of 7′′-hydroxyoleuropein took place, yielding the related 7′′-ethoxyoleuropein derivatives (Scheme 1). 

## 4. Conclusions

Fifty extracts of olive (*Olea europaea* L., cultivar Picual) wood have been prepared following seven different solvent extraction protocols in order to find the best conditions to optimize yield and radical scavenging activity. It was observed that the yields of the ethanolic, aqueous, and acid-aqueous extracts were higher than those of the ethyl acetate extracts, while the opposite was observed for the antioxidant activity. Indeed, the most active extracts were obtained with ethyl acetate either through direct extraction or by successive liquid-liquid partitioning procedures. When the extracts were obtained under reflux, the yields were higher than at room temperature, although the radical scavenging activities were similar. There are no significant differences between the results obtained from the pretreatment of olive wood with sulfuric acid or hydrochloric acid, neither in aqueous-alcoholic solutions nor in aqueous solutions. Pretreatment times of 3 and 5 h gave higher radical scavenging activities than those of 1 h. The best result for the hydrolysis pretreatments (with yields of 9.0 g extract kg^−1^ wood and radical scavenging percentages of 68.7%) was similar to that obtained for ethyl acetate extractions without pretreatment (from 8.6 to 14.2 g extract kg^−1^ wood for yield and from 48.5 to 64.9% for radical scavenging activity). Significant differences were observed for the extraction yields and radical scavenging activity from those olive wood samples collected at two different geographical origins, years, and seasons. The HPLC–DAD–MS analysis of the ethyl acetate and ethanol extracts showed similar profiles, where oleuropein and ligustroside were the main components. However, the chromatogram of the ethanolic extract from the sample collected during the olives' harvesting season (extract **D**2) showed four major peaks: oleuropein, ligustroside, and two compounds identified as (7′′*R*)-7′′-ethoxyoleuropein (**1**) and (7′′*S*)-7′′-ethoxyoleuropein (**2**). Compounds **1** and **2** were shown to be artefacts formed from the natural product 7′′-hydroxyoleuropein during the extraction process with ethanol.

## Figures and Tables

**Table 1:** NMR data (400 MHz, CD_3_OD) of (7′′*R*)-7′′-ethoxyoleuropein (**1**) and (7′′*S*)-7′′-ethoxyoleuropein (**2**).

Position	**1**		**2**	
	*δ*_*H*_, mult. (*J* in Hz)	*δ*_*C*_, mult.	*δ*_*H*_, mult. (*J* in Hz)	*δ*_*C*_, mult.
1	5.92, bs	95.0, CH	5.92, bs	95.1, CH
3	7.51, s	155.2, CH	7.52, s	155.2, CH
4	—	109.3, qC	—	109.4, qC
5	3.99, dd (4.5, 9.0)	31.8, CH	3.94–4.00, m	31.7, CH
6a	2.72, dd (4.5, 14.2)	41.1, CH_2_	2.72, dd (4.4, 14.4)	41.1, CH_2_
6b	2.47, dd (9.0, 14.2)		2.47, dd (9.1, 14.4)	
7	—	173.1, qC	—	173.0, qC
8	6.08, bq (6.9)	124.9, CH	6.09, bq (6.6)	124.9, CH
9	—	130.5, qC	—	130.5, qC
10	1.69 bd (6.9)	13.6, CH_3_	1.69, bd (6.6)	13.6, CH
11	—	168.7, qC	—	168.7, qC
OCH_3_	3.71, s	51.9, CH_3_	3.71, s	51.9, CH_3_
1′	4.80, d (7.8)	100.8, CH	4.80, d (7.9)	100.8, CH
2′	3.34–3.42, m	74.8, CH	3.34–3.44, m	74.8, CH
3′	3.34–3.42, m	77.9, CH	3.34–3.44, m	77.9, CH
4′	3.34–3.42, m	71.5, CH	3.34–3.44, m	71.5, CH
5′	3.34–3.42, m	78.4, CH	3.34–3.44, m	78.4, CH
6′a	3.89, bd (11.9)	62.7, CH_2_	3.89, bd (11.8)	62.7, CH_2_
6′b	3.67, dd (4.9, 11.9)		3.66, dd (4.9, 11.8)	
1′′	—	131.3, qC	—	131.4, qC
2′′	6.77, bs	114.9, CH	6.77, bs	114.8, CH
3′′	—	146.5, qC	—	146.6, qC
4′′	—	146.4, qC	—	146.4, qC
5′′	6.75, d (7.6)	116.3, CH	6.75, d (8.3)	116.3, CH
6′′	6.65, bd (7.6)	119.7, CH	6.65, bd (8.3)	119.7, CH
7′′	4.39, dd (4.1, 7.2)	80.7, CH	4.39, dd (4.0, 7.8)	80.6, CH
8′′a	4.02–4.10, m	69.3, CH_2_	4.14, dd (7.8, 11.2)	69.2, CH_2_
8′′b	4.02–4.10, m		3.94–4.00, m	
OCH_2_CH_3_	3.34–3.42, m	65.2, CH_2_	3.34–3.44, m	65.2, CH_2_
OCH_2_ CH_3_	1.15, t (7.0)	15.6, CH_3_	1.15, t (7.0)	15.6, CH_3_

**Table 2:** Extraction yields and radical scavenging percentages of several extracts prepared by the extraction procedures E1–E6 from the olive wood sample **A**.

Extract^a^	Procedure^b^	Solvent	Temperature	Yield^c^	RSP ± SD^d^
**A**1	E1	EtOH	r.t.	54.1	48.8 ± 0.2

**A**2	E2	EtOAc	r.t.	11.0	63.2 ± 0.8
**A**3	E2	EtOH	r.t.	51.4	42.1 ± 0.4

**A**4	E3	EtOAc	Reflux	14.2	64.9 ± 0.1

**A**5	E4	EtOAc	r.t.	8.6	48.5 ± 0.8
**A**6	E4	EtOH	r.t.	40.0	42.1 ± 1.1
**A**7	E4	EtOH : H_2_O 3 : 2	r.t.	111.4	27.1 ± 1.9
**A**8	E4	H_2_O	r.t.	80.0	17.1 ± 1.4
**A**9	E4	H_2_O : HCOOH 4 : 1	r.t.	122.9	25.9 ± 0.5
**A**10	E4	EtOAc	Reflux	11.4	50.9 ± 1.5
**A**11	E4	EtOH	Reflux	94.3	52.4 ± 1.7
**A**12	E4	EtOH : H_2_O 3 : 2	Reflux	145.7	39.6 ± 1.1
**A**13	E4	H_2_O	Reflux	108.6	44.0 ± 0.2
**A**14	E4	H_2_O : HCOOH 4 : 1	Reflux	211.4	33.7 ± 1.8

**A**15	E5	EtOAc	Reflux	22.6	54.3 ± 0.3
**A**16	E5	*n*-BuOH	Reflux	54.9	45.7 ± 1.3
**A**17	E5	H_2_O	Reflux	42.0	11.1 ± 0.8

**A**18	E6	Et_2_O	Reflux	4.0	48.5 ± 0.1
**A**19	E6	CHCl_3_	Reflux	4.0	26.1 ± 0.4
**A**20	E6	*n*-BuOH	Reflux	33.7	39.6 ± 0.1

Rosemary oleoresin (reference extract)^d^					95.0 ± 0.3

^a^Extracts **A**1–**A**20 were prepared from the olive wood sample **A**, collected in April, 2003 (during the pruning period) at the village of Fuensanta, Jaén province, Spain.

^b^Procedures E1–E6 are detailed in Figures 1–6.

^c^Yield is expressed as grams of extract per kilogram of olive wood sample.

^d^Radical scavenging percentage (RSP) is expressed as DPPH^•^ scavenging (%). Values are means of three replicates ± SD (standard deviation).

^d^Commercially available rosemary extract was used as reference, at the same concentration (50 *μ*g mL^−1^).

**Table 3:** Extraction yields and radical scavenging percentages of several extracts prepared by the extraction procedure E7 from the olive wood sample **A**.

Extract^a^	Pretreatment^b^	Time^c^	Reextracted material^d^	Yield^e^	RSP ± SD^f^
**A**21	H_2_SO_4_ in H_2_O	1 h	Acidic extract	29.2	49.7 ± 0.6
**A**22	H_2_SO_4_ in H_2_O	1 h	Pre-treated wood	8.8	37.4 ± 0.1
**A**23	H_2_SO_4_ in H_2_O	3 h	Acidic extract	23.6	49.6 ± 0.8
**A**24	H_2_SO_4_ in H_2_O	3 h	Pre-treated wood	4.7	59.6 ± 0.4
**A**25	H_2_SO_4_ in H_2_O	5 h	Acidic extract	34.8	49.2 ± 0.9
**A**26	H_2_SO_4_ in H_2_O	5 h	Pre-treated wood	5.5	59.6 ± 1.6

**A**27	H_2_SO_4_ in H_2_O : EtOH	1 h	Acidic extract	25.7	54.0 ± 1.3
**A**28	H_2_SO_4_ in H_2_O : EtOH	1 h	Pre-treated wood	5.2	29.9 ± 1.9
**A**29	H_2_SO_4_ in H_2_O : EtOH	3 h	Acidic extract	71.2	48.1 ± 2.9
**A**30	H_2_SO_4_ in H_2_O : EtOH	3 h	Pre-treated wood	5.9	38.4 ± 1.7
**A**31	H_2_SO_4_ in H_2_O : EtOH	5 h	Acidic extract	74.5	45.7 ± 1.2
**A**32	H_2_SO_4_ in H_2_O : EtOH	5 h	Pre-treated wood	2.3	63.1 ± 0.6

**A**33	HCl in H_2_O	1 h	Acidic extract	27.5	56.6 ± 0.5
**A**34	HCl in H_2_O	1 h	Pre-treated wood	4.9	38.1 ± 0.9
**A**35	HCl in H_2_O	3 h	Acidic extract	26.4	58.5 ± 2.1
**A**36	HCl in H_2_O	3 h	Pre-treated wood	6.1	68.7 ± 1.1
**A**37	HCl in H_2_O	5 h	Acidic extract	23.5	48.8 ± 0.4
**A**38	HCl in H_2_O	5 h	Pre-treated wood	9.0	64.5 ± 0.4

**A**39	HCl in H_2_O : EtOH	1 h	Acidic extract	40.0	43.0 ± 1.2
**A**40	HCl in H_2_O : EtOH	1 h	Pre-treated wood	7.5	53.8 ± 0.3
**A**41	HCl in H_2_O : EtOH	3 h	Acidic extract	58.5	41.6 ± 0.8
**A**42	HCl in H_2_O : EtOH	3 h	Pre-treated wood	7.1	56.4 ± 1.8
**A**43	HCl in H_2_O : EtOH	5 h	Acidic extract	94.1	45.9 ± 2.4
**A**44	HCl in H_2_O : EtOH	5 h	Pre-treated wood	3.6	60.9 ± 1.9

Rosemary oleoresin (reference extract)^g^					95.0 ± 0.3

^a^Extracts **A**21–**A**44 were prepared from the olive wood sample **A**, collected in April, 2003 (during the pruning period) at the village of Fuensanta, Jaén province, Spain.

^b^The olive wood sample **A** was subjected to a hydrolysis pre-treatment with 0.5 M H_2_SO_4_ in H_2_O (extracts **A**21–**A**26), 0.5 M H_2_SO_4_ in H_2_O : EtOH (50 : 50, v/v) (extracts **A**27–**A**32), 1 M HCl in H_2_O (extracts **A**33–**A**38), and 1 M HCl in H_2_O : EtOH (50 : 50, v/v) (extracts **A**39–**A**44) (see Figure 7).

^c^Time of the hydrolysis pre-treatment on the olive wood sample **A** at 130°C.

^d^The hydrolysis pre-treatment of olive wood sample **A** afforded a liquid acidic extract and a solid pre-treated wood on which further extractions were performed (see Figure 7).

^e^Yield is expressed as grams of extract per kilogram of olive wood sample.

^f^Radical scavenging percentage (RSP) is expressed as DPPH^•^ scavenging (%). Values are means of three replicates ± SD (standard deviation).

^g^Commercially available rosemary extract was used as reference, at the same concentration (50 *μ*g mL^−1^).

**Table 4:** Extraction yields and radical scavenging percentages of several extracts prepared by the extraction procedures E3 and E4 from olive wood samples **A**, **B**, **C**, and **D**.

Olive wood sample^a^	Extract^a^	Procedure^b^	Solvent	Yield^c^	RSP ± SD^d^
**A**	**A**4	E3	EtOAc	14.2	64.9 ± 0.1
**B**	**B**1	E3	EtOAc	46.8	63.7 ± 1.3
**C**	**C**1	E3	EtOAc	91.8	59.1 ± 2.6
**D**	**D**1	E3	EtOAc	14.2	40.5 ± 0.7

**A**	**A**11	E4	EtOH	94.3	52.4 ± 1.7
**B**	**B**2	E4	EtOH	117.3	38.3 ± 2.6
**C**	**C**2	E4	EtOH	172.5	42.4 ± 0.6
**D**	**D**2	E4	EtOH	81.7	42.9 ± 2.2

Rosemary oleoresin (reference extract)^e^					95.0 ± 0.3

^a^Extracts were prepared from (a) the olive wood sample **A**, collected in April, 2003 (during the pruning period) at the village of Fuensanta, Jaén province, Spain; (b) the olive wood sample **B**, collected in March, 2005 (during the pruning period) at the same location of sample **A**; (c) the olive wood sample **C**, collected in March, 2005 (during the pruning period) at the village of Mogón, Jaén province, Spain; (d) the olive wood sample **D**, collected in November, 2006 (during the harvesting season) at the same location of sample **A**.

^b^Procedures E3 and E4 are detailed in Figures 3 and 4.

^c^Yield is expressed as grams of extract per kilogram of olive wood sample.

^d^Radical scavenging percentage (RSP) is expressed as DPPH^•^ scavenging (%). Values are means of three replicates ± SD (standard deviation).

^e^Commercially available rosemary extract was used as reference, at the same concentration (50 *μ*g mL^−1^).
